# Prevalence of natural feline coronavirus infection in domestic cats in Fujian, China

**DOI:** 10.1186/s12985-023-02273-y

**Published:** 2024-01-03

**Authors:** Bo Dong, Xiaodong Zhang, Xiaowei Zhong, Wenqian Hu, Zhihui Lin, Shuo Zhang, Haiyan Deng, Weiming Lin

**Affiliations:** 1 College of Life Science of Longyan University, 364012 Longyan, China; 2 Engineering Research Center for the Prevention and Control of Animal Original Zoonosis, College of Life Science, Fujian Province University, Longyan University, Longyan, China; 3 Fujian Provincial Key Laboratory for the Prevention and Control of Animal Infectious Diseases and Biotechnology, Longyan, China

**Keywords:** Feline coronaviruses, Prevalence, Epidemiology, Faeces samples, RT-PCR

## Abstract

Only few studies have investigated the prevalence of feline coronavirus (FCoV) infection in domestic cats in Fujian, China. This is the first study to report the prevalence rate of FCoV infection in domestic cats in Fujian, China, and to analyse the epidemiological characteristics of FCoV infection in the region. A total of 112 cat faecal samples were collected from animal hospitals and catteries in the Fujian Province. RNA was extracted from faecal material for reverse transcription polymerase chain reaction (RT-PCR). The prevalence rate of FCoV infection was determined, and its epidemiological risk factors were analysed. The overall prevalence of FCoV infection in the cats, was 67.9%. We did not observe a significant association between the age, sex, or breed of the cats included in the study and the prevalence rate of the viral infection. Phylogenetic analysis showed that the four strains from Fujian were all type I FCoV. This is the first study to analyse the prevalence and epidemiological characteristics of FCoV infection in domestic cats in Fujian, China, using faecal samples. The results of this study provide preliminary data regarding the prevalence of FCoV infection in the Fujian Province for epidemiological studies on FCoV in China and worldwide. Future studies should perform systematic and comprehensive epidemiological investigations to determine the prevalence of FCoV infection in the region.

**Supplementary Information:**

The online version contains supplementary material available at 10.1186/s12985-023-02273-y.

## Introduction

Feline coronavirus (FCoV) is a non-segmented, single-stranded RNA virus; it belongs to the *Alphacoronavirus* family [1]. Based on its pathogenicity, FCoVs are divided into two biotypes: feline enteric coronavirus (FECV) and feline infectious peritonitis virus (FIPV) [2]. FCoV infections are common in domestic and wild cats of all ages, and the virus is mainly transmitted through the faecal–oral route. FECV infection does not exhibit any obvious systemic clinical manifestations, whereas FIPV infection can cause peritonitis or nervous system damage and infectious peritonitis (FIP) in cats with high mortality. Mutations in the FCoV genome play an important role in the development of FIP. These mutations are responsible for the changes in viral tropism from intestinal cells to monocytes/macrophages. The mutations in the S gene of FCoVs play an important role in the changes in viral tropism and pathogenicity. Thus, FIPV is highly virulent, leading to the development of fatal FIP, which is characterised by fibrinous peritonitis, massive accumulation of ascites and high mortality rate. However, effective methods to prevent or control FIPV infection are not available.

Based on the difference in the amino acid sequence of the S protein, FCoV can be divided into two genotypes: type I and type II [3], and both genotypes have been reported in FECVs and FIPVs. In most countries, FCoV type I strains exhibit higher infection rate than FCoV type II strains [4, 5]. Moreover, in China, FCoV type I strains are highly prevalent in both domestic and healthy cat populations with FIP [6, 7]. However, the main clinical sign of type I or type II FECV infections is mild enteritis, which is not considered a serious issue, and no signs remain undetected. However, once FECV transforms into FIPV because of mutations, the virus spreads systemically with increased infectivity, leading to harmful clinical signs and high mortality rate. FECV is transmitted through the faeces of healthy cats that are infected by direct contact with faeces or contaminated cat litter and other contaminants. Therefore, epidemiological monitoring of the presence of FCoVs in domestic cat faeces is essential to identify the characteristics of viral infection in domestic cat populations and to control FIP without effective treatment.

Reverse transcription-polymerase chain reaction (RT-PCR)-based virus detection methods are better than serological tests because they directly detect persistent viral infections instead of determining the presence of antibodies in the host serum sample based on previous contact with the coronavirus [8]. Many studies have used RT-PCR to detect FCoV in cat faeces and have demonstrated acceptable diagnostic efficacy [9–11]. However, the prevalence of FCoV infection in domestic cats in Fujian, China, and the epidemiological factors related with the infection have not been reported. Therefore, in this study, we used RT-PCR to detect FCoV in faecal samples of domestic cats in the Fujian Province. We also summarised the epidemiology of FCoV infection in domestic cats in the Fujian Province.

## Materials and methods

### Clinical sample collection

In this study, faecal samples were collected from 112 domestic cats treated in animal hospitals or kept in catteries in Fujian, China (Table 1). The personnel involved in the study removed fresh faecal particles from the litter tray, placed them in a sterile tank and sent them to the laboratory. The samples were stored at 4 °C for up to 72 h, and a 10% (w/v) phosphate buffered saline (PBS) faecal suspension was immediately prepared upon receipt and stored at -80℃. This study was approved by the Animal Experiment Ethics Committee of Longyan University. All animal experiments were performed after obtaining ethics approval from the Committee on the Ethics of Animal Experiments of Longyan University (LY2023001L). The study was conducted in compliance with the ARRIVE guidelines. Informed consent was obtained from the cat owners prior to sample collection, and the cat owners approved the sampling and data release.

**Table 1:** Summary for 112 domestic cats with feline coronavirus

Cat No	Breed	Age	Gender	Collectionn Season	RT-PCR
1	Hybrid	≥ 1 year	Male	Spring	-
2	Purebred	< 1 year	Female	Spring	+
3	Purebred	≥ 1 year	Male	Spring	+
5	Hybrid	≥ 1 year	Female	Spring	-
6	Hybrid	≥ 1 year	Male	Spring	-
9	Purebred	≥ 1 year	Male	Spring	-
10	Purebred	≥ 1 year	Female	Spring	+
11	Purebred	≥ 1 year	Male	Spring	+
12	Purebred	≥ 1 year	Female	Spring	-
13	Purebred	≥ 1 year	Male	Spring	+
15	Purebred	≥ 1 year	Female	Spring	-
16	Hybrid	≥ 1 year	Female	Spring	+
17	Hybrid	< 1 year	Male	Spring	+
18	Hybrid	< 1 year	Male	Spring	-
20	Hybrid	< 1 year	Female	Spring	+
22	Purebred	≥ 1 year	Female	Spring	-
23	Hybrid	≥ 1 year	Male	Spring	+
24	Purebred	≥ 1 year	Female	Spring	+
25	Purebred	≥ 1 year	Male	Spring	+
26	Purebred	≥ 1 year	Female	Spring	+
27	Hybrid	≥ 1 year	Female	Spring	-
28	Hybrid	< 1 year	Male	Spring	+
29	Hybrid	< 1 year	Male	Spring	+
30	Hybrid	≥ 1 year	Male	Spring	-
31	Hybrid	≥ 1 year	Male	Spring	-
32	Hybrid	≥ 1 year	Female	Spring	-
33	Hybrid	< 1 year	Male	Spring	+
34	Hybrid	< 1 year	Male	Spring	+
35	Hybrid	< 1 year	Male	Spring	-
36	Hybrid	< 1 year	Male	Spring	+
37	Hybrid	≥ 1 year	Male	Spring	-
38	Hybrid	≥ 1 year	Female	Spring	+
40	Hybrid	≥ 1 year	Male	Spring	-
42	Purebred	< 1 year	Female	Spring	-
43	Purebred	≥ 1 year	Male	Spring	-
44	Purebred	≥ 1 year	Male	Spring	+
45	Purebred	≥ 1 year	Female	Spring	+
46	Purebred	≥ 1 year	Male	Spring	+
47	Hybrid	≥ 1 year	Male	Spring	-
51	Hybrid	≥ 1 year	Female	Spring	-
52	Purebred	< 1 year	Female	Spring	-
54	Purebred	< 1 year	Female	Spring	+
58	Hybrid	< 1 year	Male	Spring	+
59	Purebred	< 1 year	Female	Spring	+
60	Hybrid	< 1 year	Female	Spring	+
62	Purebred	≥ 1 year	Female	Spring	+
63	Purebred	≥ 1 year	Male	Spring	+
65	Purebred	≥ 1 year	Male	Spring	+
66	Purebred	≥ 1 year	Male	Spring	+
67	Hybrid	< 1 year	Female	Spring	+
69	Purebred	≥ 1 year	Female	Spring	-
70	Purebred	≥ 1 year	Female	Spring	+
71	Purebred	< 1 year	Male	Spring	-
73	Purebred	< 1 year	Female	Spring	+
74	Purebred	< 1 year	Male	Spring	+
75	Purebred	< 1 year	Female	Spring	+
84	Purebred	< 1 year	Male	Summer	+
86	Purebred	< 1 year	Female	Summer	-
87	Hybrid	< 1 year	Male	Summer	+
88	Purebred	< 1 year	Male	Summer	+
89	Purebred	< 1 year	Female	Summer	+
90	Purebred	< 1 year	Male	Summer	-
93	Hybrid	≥ 1 year	Male	Summer	+
94	Purebred	≥ 1 year	Male	Summer	+
95	Purebred	≥ 1 year	Male	Summer	-
97	Hybrid	≥ 1 year	Male	Summer	-
98	Hybrid	≥ 1 year	Female	Summer	-
99	Hybrid	< 1 year	Male	Summer	-
102	Hybrid	≥ 1 year	Female	Autumn	+
103	Purebred	≥ 1 year	Male	Autumn	+
104	Hybrid	≥ 1 year	Male	Autumn	+
105	Purebred	≥ 1 year	Male	Autumn	+
118	Purebred	≥ 1 year	Male	Winter	+
119	Purebred	≥ 1 year	Male	Winter	+
120	Hybrid	≥ 1 year	Female	Winter	+
121	Hybrid	≥ 1 year	Female	Winter	+
122	Hybrid	≥ 1 year	Female	Winter	+
123	Hybrid	≥ 1 year	Male	Winter	+
124	Purebred	≥ 1 year	Male	Winter	-
125	Hybrid	≥ 1 year	Female	Winter	+
126	Purebred	< 1 year	Male	Winter	+
127	Hybrid	≥ 1 year	Male	Winter	-
129	Hybrid	≥ 1 year	Male	Spring	-
130	Hybrid	≥ 1 year	Female	Spring	+
131	Purebred	≥ 1 year	Male	Spring	-
133	Hybrid	≥ 1 year	Male	Spring	+
134	Hybrid	≥ 1 year	Female	Spring	-
135	Purebred	< 1 year	Female	Spring	-
136	Purebred	< 1 year	Female	Spring	+
137	Purebred	≥ 1 year	Male	Spring	+
138	Hybrid	≥ 1 year	Male	Spring	+
139	Purebred	≥ 1 year	Male	Spring	+
140	Purebred	≥ 1 year	Female	Spring	+
141	Purebred	≥ 1 year	Male	Spring	-
144	Purebred	≥ 1 year	Male	Spring	-
145	Purebred	≥ 1 year	Female	Spring	+
147	Hybrid	≥ 1 year	Male	Spring	+
148	Purebred	≥ 1 year	Female	Spring	+
152	Purebred	≥ 1 year	Male	Spring	+
153	Hybrid	≥ 1 year	Male	Spring	+
156	Purebred	≥ 1 year	Female	Spring	+
157	Purebred	< 1 year	Female	Spring	+
158	Purebred	≥ 1 year	Female	Spring	+
159	Purebred	≥ 1 year	Male	Spring	+
160	Purebred	≥ 1 year	Female	Spring	+
161	Purebred	≥ 1 year	Male	Spring	+
162	Purebred	≥ 1 year	Female	Spring	+
163	Purebred	≥ 1 year	Male	Spring	+
164	Hybrid	≥ 1 year	Male	Spring	+
165	Hybrid	≥ 1 year	Male	Spring	-
166	Hybrid	≥ 1 year	Male	Spring	+
167	Purebred	≥ 1 year	Female	Spring	+

+: The PCR results were positive, -: The PCR results were negative

### RT-PCR

Total RNA was extracted from 200 µL of the faecal suspensions using the Simply P Virus RNA Extraction Kit (Hangzhou Bioer Technology Co. Ltd, China). RT-PCR was performed at 42℃ for 1 h using 3 µg of total RNA, 0.5 µg of random primers (Promega, Madison, WI, USA) and 5 U AMV reverse transcriptase (Promega). RT-PCR was performed to amplify a highly conserved FCoV N-gene sequence, as per a previously described method [6].

### Epidemiological survey and statistical analysis

A total of 112 faecal samples were collected from cats at animal hospitals in Fujian in 2022. The samples were tested for FCoV using RT-PCR. The overall prevalence rate was calculated based on detection results. Moreover, epidemiological factors were classified according to the age, sex and breed. All data were analysed using the GraphPad Prism 8.0 software (GraphPad Software, San Diego, CA, USA). The prevalence rates in the two groups were compared using the χ^2^ test. Statistical significance was set at *P* < 0.05.

### Cloning and sequencing

In order to identify the phylogenetic characteristics of FCoV strains from Fujian, full-length N genes from 4 positive samples (FJFZ01, FJLY01, FJLY02, FJLY05) were amplified, cloned and sequenced according to previous reports [6]. The amplified PCR products were subjected to gel electrophoresis, excised, and purified using an agarose gel DNA purification kit (Takara Biomedical Technology (Beijing) Co. Ltd, China). The purified PCR products were sent to Shanghai Sangon Biological Engineering Technology and Services Co., Ltd. (Shanghai, China) for sequencing.

### Phylogenetic analysis

The nucleotide sequences of Fujian strains were compared with *N gene* sequences from other retrieved FCoV strains previously published in GenBank (Table 2). Multiple sequence alignment and sequence analysis were performed using the Multiple Sequence Alignment tool of the DNAMAN 6.0 software (Lynnon BioSoft, Point-Claire, Quebec, Canada). Sequences across different viral strains were compared using the pairwise distances of the untitled ClustalW (weighted) method. Phylogenetic trees derived from the nucleotide sequences were constructed by MEGA version 5.2 using the neighbor‐joining method with the p‐distance model, 1,000 bootstrap replicates.

**Table 2:** The information of the reference strains in this study

Strain name	ID number	Year	Country	Type I or II
FCoV strain HLJ DQ 2016 01	KY292377	2016	China	type I
FCoV strain HLJ HRB 2016 16	KY566208	2016	China	type I
FCoV strain HLJ DQ 2016 05	KY566207	2016	China	type I
FCoV black	EU186072	2007	USA	type I
FCoV strain NLD UU88 2010	KF530123	2010	Netherlands	type I
FCoV UU54	JN183883	2010	Netherlands	type I
FECV-UCD5	FJ917522	2008	USA	type I
FECV-UCD4	FJ943763	2008	USA	type I
FECV-UCD3a	FJ943761	2007	USA	type I
FIPV 79-1146	DQ010921	2005	UK	type II
FIPV	NC_002306	2005	USA	type II
FIPV strain DF-2	JQ408981	2012	Hungary	type II

## Results

### Detection and analysis of FCoV in Fujian

Our analysis revealed that 67.9% (76/112) of the feline faecal samples tested positive for FCoV (Table 3). Furthermore, we observed 66.2 and 70.2% prevalence of FCoV infection in faecal samples from male (43/65) and female cats (33/47), respectively. A total of 80 and 32 cats included in the study were aged > 1 and < 1 year, respectively, with 66.3% (53/80) and 71.9% (23/32) prevalence of FCoV infection, respectively. According to the breed classification, 63 faecal samples belonged to purebred cats, with 73.0% (46/63) prevalence of FCoV infection, and 49 were hybrid cats, with 61.2% prevalence (30/49).

**Table 3:** Prevalence rate of feline coronavirus (FCoV) infection based on season and age, breed and sex of cats

Risk factor		No. of FCoV-positive cats	No. cats of examined	Prevalence rate (%)	*P* value
**Age**	< 1 year	23	32	71.9	0.56^*^
	≥ 1 year	53	80	66.3	
**Breed**	Purebred	46	63	73.0	0.19^†^
	Hybrid	30	49	67.2	
**Sex**	Male	43	65	66.2	0.65^‡^
	female	33	47	70.2	

*: Compared with ‘1 year of age or above’, †: Compared with ‘hybrid’, ‡: Compared with ‘female’

### Phylogenetic analysis

**Fig. 1:**
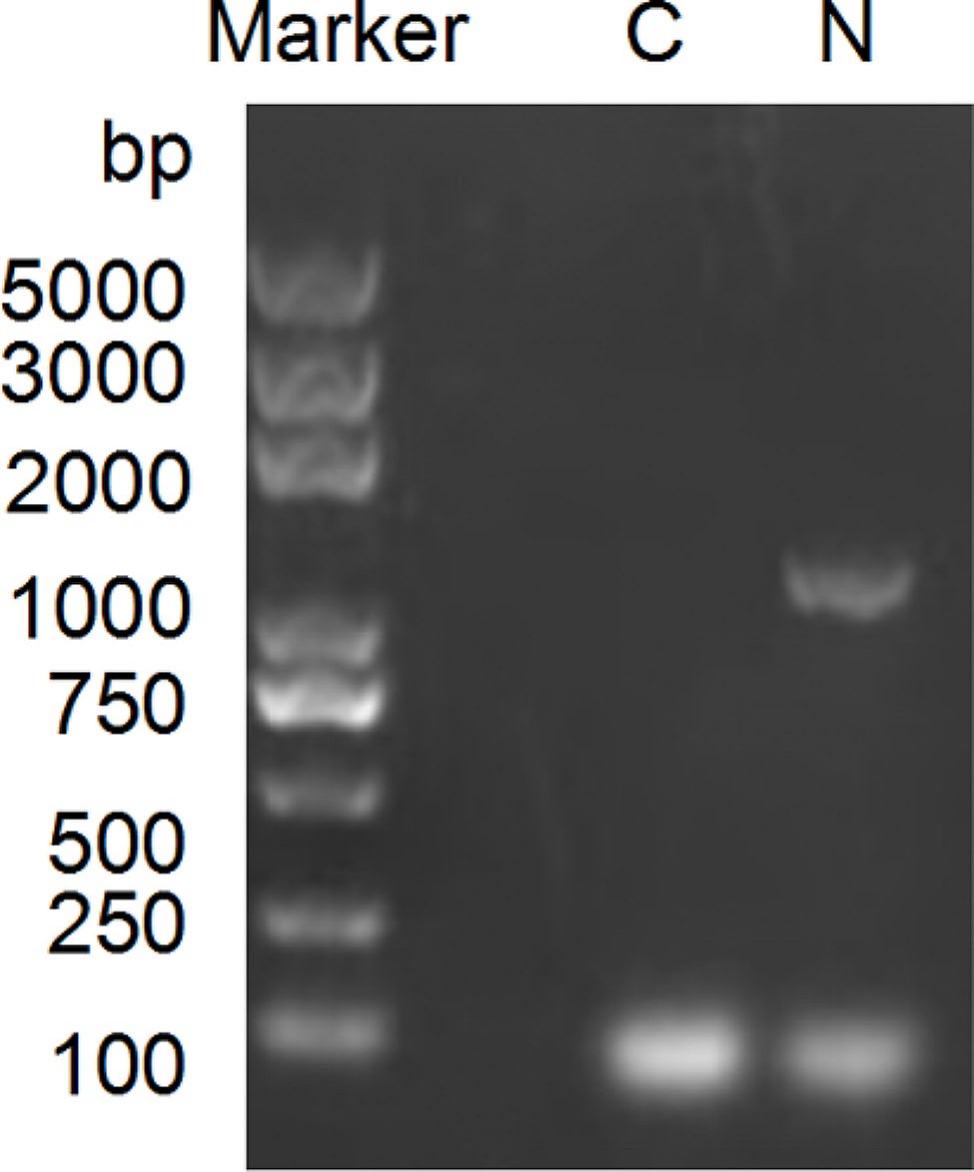
Amplification of N gene. Amplification of 1134 bp (bp) DNA fragment from the ascitic fluid specimen. Marker: 5000 bp molecular weight ladder, C: negative control, N: PCR product of N gene

**Fig. 2:**
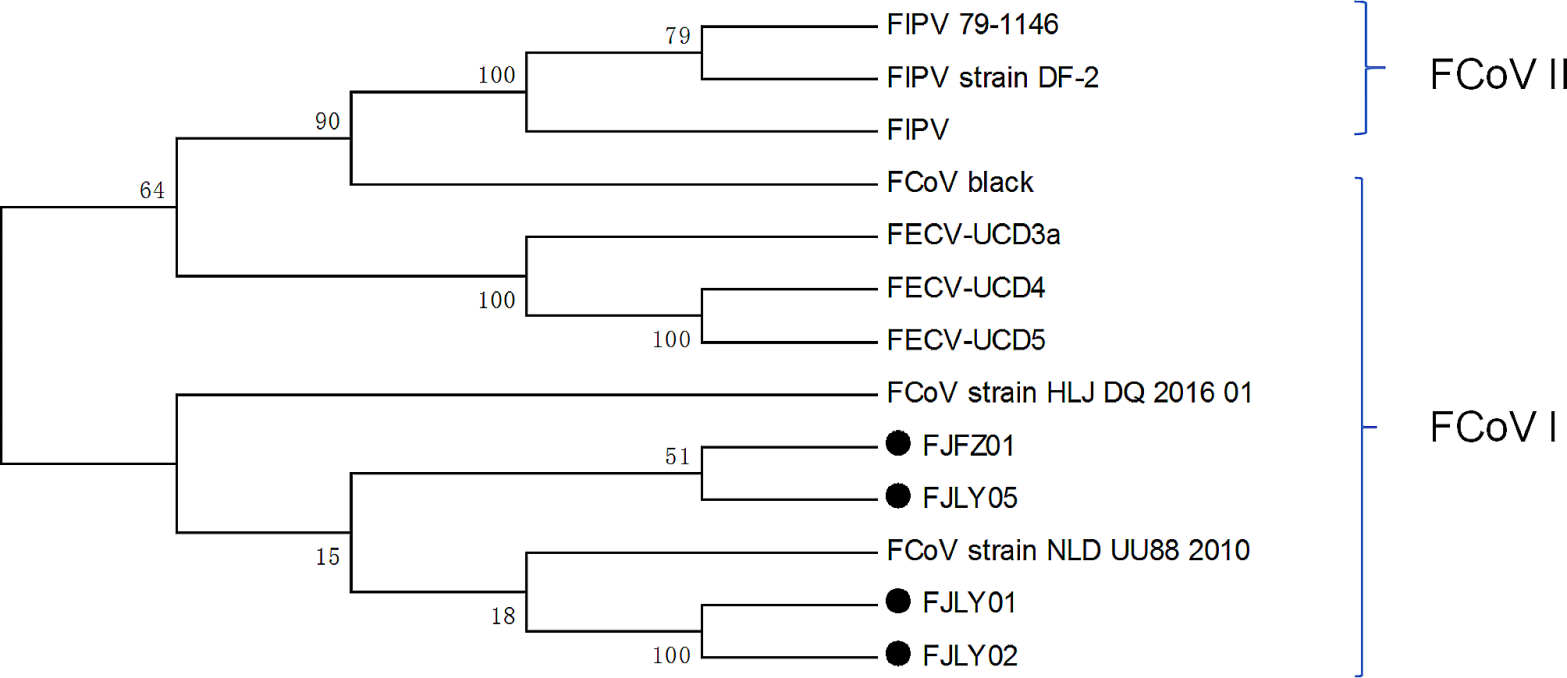
N genes phylogenetic analysis. The phylogenetic tree was generated using the neighbor-joining method, and supported by 1000 bootstraps. The ● represent identified Fujian strains in our study

The N gene amplification of fecal samples from FCoV-positive cats resulted in the expected 1134 bp product (Fig. 1). Four complete N genes were obtained from four strains in Fujian, China. The results of multiple sequence comparison showed that the nucleotide sequence identity of Fujian strains was 91.6–99.5%. The identity between Fujian strain and other strains collected in this study was 89.2-91.5%. The identity of Fujian strain with FCoV-Black was 90.0-91.9%, and the identity with FCoV II strain (WSU 79-1146) was 89.4–91.4%. Phylogenetic analysis showed that compared with type I and type II FCoV strains, the Fujian strains were located in type I FCoV strains cluster (Fig. 2).

## Discussion

FIP is a highly prevalent infectious disease in the cat population of China, but an effective vaccine or a specific treatment against FIP is not available. Therefore, the epidemiological characteristics of FCoV in this region should be investigated to prevent FIP. High prevalence of digestive tract colonisation by FCoV has been reported in the cat populations of different countries, including Portugal (47.5%) [12], Germany (76.5%) [13], Malaysia (84%) [14] and Japan (37%) [15]. High prevalence of FCoV infection among cat populations in some areas of China has also been reported [7]. These data provide information to understand the prevalence of FCoV infection in China. However, in China, the climate exhibits regional variation; thus, the epidemiological characteristics of FCoV infection in all areas of China have not been elucidated. In our previous study, we collected serum samples from domestic cats in Fujian, China, and found a FCoV-positive rate of 70.09% [16]. However, positive antibody test results could also be because of the presence of maternal antibodies. PCR-based tests directly detect the FCoV genome instead of determining the presence of antibodies against the coronavirus in the serum [14]. Therefore, PCR-based detection using faecal samples is more indicative of FCoV infection in cats. To the best of our knowledge, this is the first report of FCoV detection in the faecal samples collected from a cat population of Fujian. The results revealed that the prevalence of FCoV infection in the cat population was 67.9%, indicating that FCoV infection is highly prevalent in Fujian; the prevalence rate is similar to that in the other provinces of China, thus measures should be taken to prevent the infection.

Although younger animals are more susceptible to the coronavirus [17, 18], the results of this study revealed that cats of all ages can be infected with FCoV, and a significant difference in the infection rate between the cats of different ages was not observed. Moreover, we observed slightly higher prevalence rate of FCoV infection in cats aged < 1 year than in those aged > 1 year. This may be because young cats are less resistant to disease and may be subjected to more stress from external factors, such as weaning; change in food, owner and/or habitat; separation from the parent cat; and transportation. These factors induce stress responses in young cats, which increase their susceptibility to infection. Moreover, kittens in catteries are often raised in groups before sale, which is more likely to cause FCoV infection in individual kittens.

In China, catteries for breeding purposes mainly raise female cats, which may suffer more stress than male cats due to oestrus, mating, breastfeeding and other physiological processes. Therefore, this may be the reason for the slightly higher prevalence rate of FCoV infection in female cats than in male cats in the present study. Additionally, inbreeding of purebred cats is done in some catteries in China. The purified cats exhibit poor disease resistance and environmental adaptation, which may explain the high prevalence rate of FCoV infection in purebred cats in the present study.

According to serological characteristics, FCoV can be divided into type I and type II [3]. The type I FCoV strains are more common clinically. In Europe and the United States, the prevalence of type I FCoV infection is as high as 80–95% [4, 5]. In addition, the type I FCoV strain also has a high prevalence in the Chinese cat population [6, 7]. In this study, four Fujian strains were found to be type I FCoV, indicating that type I FCoV is a subtype of infection virus prevalent in domestic cats in China, consistent with other reports.

The study has some limitations. One of the driving factors for viral infections is the season. The virus can proliferates and spreads during favourable seasons. However, owing to limited daily activities due to coronavirus disease 2019 (COVID-19), the low number of faecal samples collected in fall and winter, so the prevalence varied with season throughout the year was not shown. Therefore, seasonal variations in the prevalence rate of FCoV infection require further investigation. Moreover, further research with a larger sample size is required to determine the overall prevalence rate and epidemiological pattern of FCoV infection in cats living in the Fujian Province.

## Conclusions

This epidemiological study elucidated that 67.9% of domestic cats living in Fujian were infected with FCoV. This high prevalence requires more attention on FCoV prevention and control in domestic cats in Fujian Province, China. However, significant differences in the prevalence rate of FCoV infection were not observed between young and adult cats, male and female cats or purebred and hybrid cats.

### Electronic supplementary material

Below is the link to the electronic supplementary material.

Supplementary Material 1

## Data Availability

The data analyzed during the current study was available from the corresponding author on reasonable request.
