# Supplementary figures and images for: Prevalence of natural feline coronavirus infection in domestic cats in Fujian, China
Source: Virol J. 2024 Jan 3;21:2. doi: 10.1186/s12985-023-02273-y (PMC10765712; doi:10.1186/s12985-023-02273-y)

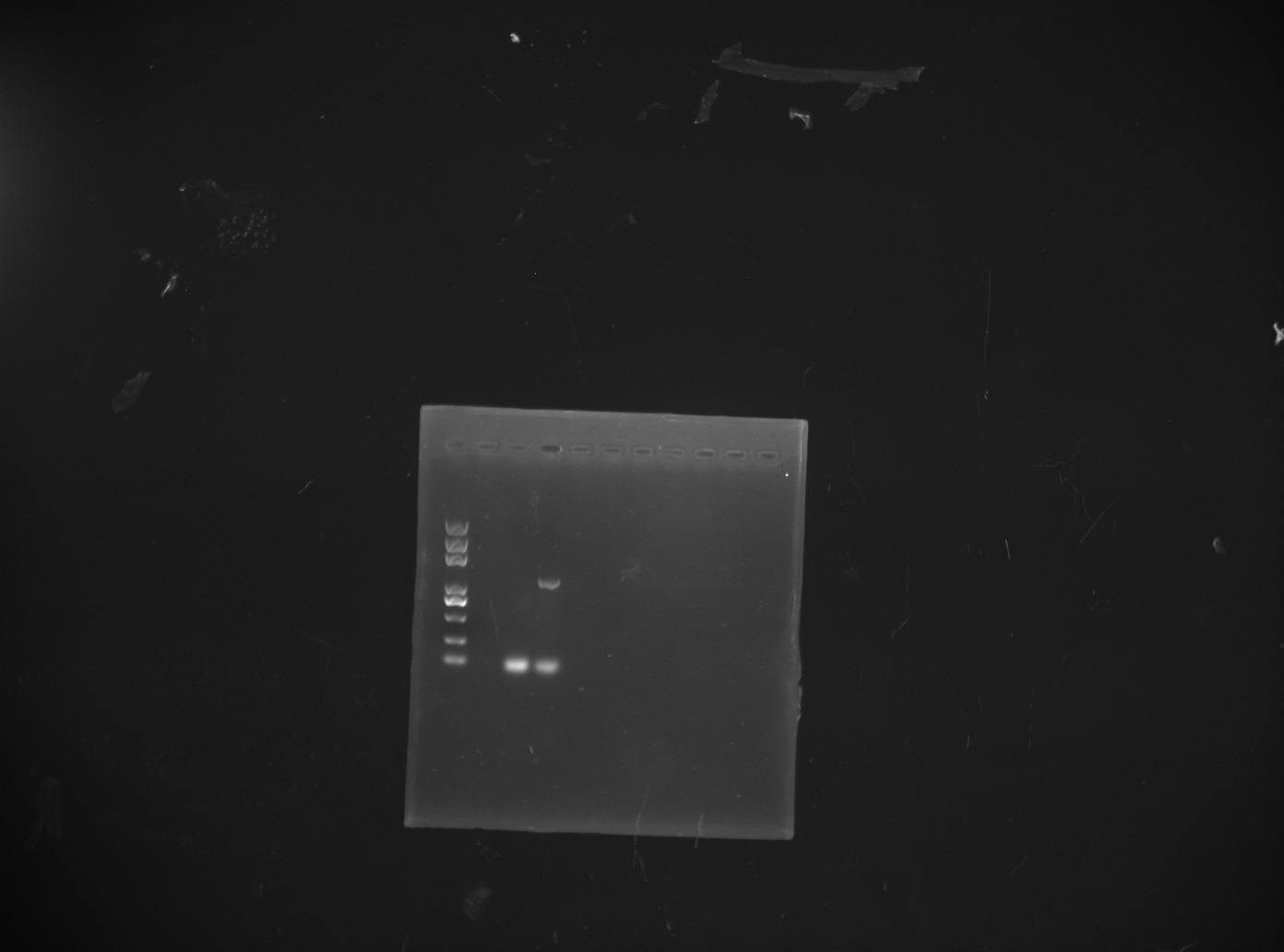

Supplement: Supplementary file 1 — Supplementary Material 1 [file 12985_2023_2273_MOESM1_ESM.jpg]
